# Qualitative research on undergraduate nursing students' recognition and response to short videos’ health disinformation

**DOI:** 10.1016/j.heliyon.2024.e35455

**Published:** 2024-07-31

**Authors:** Ming Yang, Wanyu Huang, Meiyu Shen, Juan Du, Linlin Wang, Yin Zhang, Qingshan Xia, Jingying Yang, Yingjie Fu, Qiyue Mao, Minghao Pan, Zheng Huangfu, Fan Wang, Wei Zhu

**Affiliations:** a Xinyang Central Hospital, Xinyang City, 464000, Henan Province, China; b School of Public Health, Wuhan University, Wuhan City, 430071, Hubei Province, China; c Department of Psychiatry, Renmin Hospital of Wuhan University, Wuhan, 430060, China; d School of Nursing, Fourth Military Medical University, Xi'an City, 710032, Shaanxi Province, China; e Medical College, Xinyang Normal University, Xinyang City, 464000, Henan Province, China; f Centre for Health Management and Policy Research, School of Public Health, Cheeloo College of Medicine, Shandong University, Jinan City, 250012, Shandong Province, China; g School of Information Engineering, Hubei Light Industry Technology Institute, Wuhan City, 430070, Hubei Province, China; h School of Journalism and Communication, Nanjing Xiaozhuang University, Nanjing City, 210000, Jiangsu Province, China; i School of Information Management, Wuhan University, Wuhan City, 430072, Hubei Province, China

**Keywords:** Short video health disinformation, Short video identification, Short video response, In-depth interview

## Abstract

**Background:**

With the popularity of the internet, short videos have become an indispensable tool to obtain health information. However, avoiding health disinformation owing to the openness of the Internet is difficult for users. Disinformation may endanger the health and lives of users.

**Objective:**

With a focus on the process of identifying short videos' health disinformation and the factors affecting the accuracy of identification, this study aimed to investigate the identification methods, coping strategies, and the impact of short videos’ health disinformation on undergraduate nursing students. The findings will provide guidance to users on obtaining high-quality and healthy information, in addition to reducing health risks.

**Methods:**

Semi-structured in-depth interviews were conducted with 22 undergraduate nursing students in October 2022, and data were collected for collation and content analyses.

**Results:**

The techniques used to identify short videos that include health disinformation as well as how undergraduate nursing students perceived these videos' features are among the study's findings. The failure factors in identification, coping paths, and adverse impacts of short videos on health disinformation were analyzed. The platform, the material itself, and the students' individual characteristics all have an impact on their identifying behavior.

**Conclusions:**

Medical students continue to face many obstacles in identifying and responding to health disinformation through short videos. Preventing and stopping health disinformation not only requires individual efforts to improve health literacy and maintain rational thinking, it also requires the joint efforts of short video producers, relevant departments, and platforms.

## 1 Introduction

Short videos have gained popularity as powerful engines for disseminating medical health information [1]. As of June 2022, the number of users of short videos had hit 962 million, accounting for 91.5 % of total Internet users, according to the 50th China Statistical Report on Internet Development [2]. The Internet is an essential source of health information [3,4]. Many health professionals and medical institutions have attempted to distribute health knowledge and promote public health literacy through short video applications [5]. Access to information has become increasingly varied and has an unprecedented influence on people's daily lives [6]. Through social media, users can obtain health information related to their symptoms, communicate with healthcare professionals, share their experiences, and offer support to those affected by the same disease [7].

The quality of video messages varies owing to the competence and capacity of short video producers and the lack of regulatory standards or regulations on social media [8]. Therefore, some health-related content does not meet the standards for short videos [[9], [10], [11], [12], [13]]. “Health disinformation online” has been defined as inaccurate or untrue online health information published through the Internet [14]. The motivation for disseminating disinformation is to cause harm by sharing disinformation [15]. Disinformation is nothing new and dates to the Roman Empire [[15], [16], [17]]. With the proliferation of Web 2.0 technologies, the advent of social media, including Twitter, Instagram, and WhatsApp, means that content creation is no longer limited to traditional news outlets [18,19]. In the past, YouTube also had a fairly high percentage of videos containing misleading health information [20,21]. YouTube videos related to neck pain contain low quality, low reliability, and incomplete information [22]. In 2016, more than half of the 20 most common articles on cancer on Facebook involved medical claims that were not credible [23]. Misleading information on Facebook became more popular than accurate information on the spread of disease [20]. Medical and public health professionals also expressed serious concerns regarding the quality and reliability of health information on social media [24,25].

Users find it challenging to assess the credibility and quality of health information on social media [26]. Health literacy can facilitate or complicate information-seeking, interpretation, and decision-making, thereby influencing health outcomes [27]. Users with limited health literacy and poor analytical skills may not be able to assess the accuracy of online information [28]. A study of Italian medical students revealed that before structured intervention, medical students had difficulties searching for quality health information, and most were skeptical about the usefulness of health information on the Internet [29]. YouTube users, especially novice medical clinical trainees or laypeople, do not easily recognize differences in video quality [30,31]. Previous studies have demonstrated the potential differences between consumers and primary care physicians (PCPs) in independently identifying false and misleading information in prescription drug promotions. Among consumers, exposure to deceptive claims or tactics did not increase suspicion of website veracity. Among PCPs, exposure to more deceptive claims and tactics resulted in higher perceived website deceptiveness [32].

With the increasing availability of information and communication technologies (ICTs), large amounts of disinformation can be easily disseminated to a much larger group of people at a very low cost and in a short period [26]. Health disparities have been defined as seemingly avoidable health differences among people who are socially disadvantaged [33], and one study has revealed that people facing inequitable health outcomes are relatively more likely to encounter health disinformation than more useful information [34]. Sharing among users is likely to drive the spread of disinformation on social media [35], and most users are vulnerable to social media disinformation regarding health [36]. The spread of disinformation makes the likelihood of serious adverse health outcomes high [37,38]. When disinformation or news is repeatedly disseminated, the weak influence of truthful information on a population is limited, which may influence people's health decisions, behaviors, and beliefs while encouraging unhealthy behaviors [[39], [40], [41], [42]]. For example, social media disinformation regarding COVID-19 has increased vaccine hesitancy, decreased vaccination rates, and led to preventable deaths [[43], [44], [45]]. Disinformation on social media played a role in many Iranian citizens consuming large volumes of methanol in early 2020, as they believed that this would protect them from being infected by the virus. This led to 2,000 hospital admissions for methanol poisoning and 264 subsequent deaths across the country [46]. Dramatic means are used in social networking videos to capture the short attention span of the audience, and some of these unscientific therapies and medications may alter patients' viewpoints [47]. Additionally, health information on social media can have a direct impact on people's mental health [48], and large-scale disinformation and misconceptions about health can cause public anxiety and trepidation [32,49,50]. The dissemination of health information can also lead to a reduction in patient trust in clinicians, mainstream health organizations, governments, and policymakers [51], while potentially jeopardizing the important doctor-patient relationship [14]. The dissemination of health disinformation through social media has become a major public health problem [9], and disinformation has become a significant challenge for society as a whole [17]. Social media platforms that elevate the most-viewed posts, regardless of source credibility, potentially contribute to the viral spread of disinformation. Therefore, social media platforms must provide indicators that identify and elevate credible sources of health information [52].

Social media platforms, such as TikTok, are powerful channels for young people to obtain health-related information [47]. In China, the number of active daily app users is 600 million [53]. Short videos often come with attention-grabbing images and sounds, and these videos are more difficult to review than text-based content; therefore, they are more likely to be recalled, evoke emotions, and persuade people [54]. Social networks have a great influence on young people and even change their habits and lifestyles [55,56]. Compared to other student groups, medical students are more concerned about their health [57], and their trend of using the Internet for health information is also increasing [58]. Therefore, this study investigated undergraduate nursing students’ identification methods, coping paths, and the influence of short videos on health disinformation.

## 2 Methods

### 2.1 Study design

This study conducted semi-structured interviews with undergraduate nursing students using TikTok to obtain data and explore their identification strategies, coping paths, and influences related to short videos’ health disinformation.

### 2.2 Recruitment

The interviewees were undergraduate nursing students. The interviews were completed in October 2022, and approval was obtained from the Ethics Committee of Xinyang Normal University (Study ID: XFEC-2022–21).

### 2.3 Participants

The study participants were 22 undergraduate nursing students (15 female and seven male). Out of them, 50 % (11/22) were juniors, 45 % (10/22) were sophomores, and 5 % (1/22) were freshmen. Their age averaged 20. General information on the interviewees is presented in Table 1.

**Table 1:** General information of interviewees(n = 22).

Participant	Gender	Age	The main source of short videos	Average daily viewing time of short videos(hour)	Short video viewing years	Whether there was a history of identifying failures	Attention level of short health videos
N1	female	20	TikTok	4	3	No	low
N2	female	19	TikTok	1	2	No	low
N3	female	20	TikTok	2	2	No	high
N4	female	20	TikTok	1	2	No	high
N5	female	20	TikTok	2	2	Yes	medium
N6	female	20	TikTok	3	5	No	high
N7	female	20	TikTok	1	3	No	high
N8	female	20	Quick Hand	1	3	No	medium
N9	female	19	TikTok	2	3	Yes	high
N10	female	19	TikTok	2	2	Yes	low
N11	female	19	TikTok	3	4	Yes	medium
N12	female	19	TikTok	2	4	No	high
N13	male	20	TikTok	1	3	Yes	low
N14	male	20	TikTok	3	6	Yes	high
N15	female	20	TikTok	2	4	Yes	high
N16	female	19	Little Red Book	4	4	Yes	medium
N17	male	19	TikTok	2	6	Yes	medium
N18	female	19	TikTok	0.5	1	Yes	low
N19	male	20	TikTok	1	5	No	high
N20	male	18	Quick Hand	1	4	No	low
N21	male	20	TikTok	2	3	Yes	low
N22	male	20	TikTok	3	3	Yes	high

Note.

High: browsing every day, medium: 3–6 times a week, low: less than 3 times a week.

### 2.4 Data collection

This study started with a preliminary outline of the interviews based on previous research and discussions with the medical informatics faculty. Subsequently, a final outline of the interviews was determined after pre-interviews were conducted with two individuals. The main outline of the interview was as follows: (1) “How do you access short health videos?” (2) “How do you identify short videos' health disinformation?” (3) “What do you think when you find out that it is a false health video?” (4) “What is your opinion on the spread of short videos' health disinformation?” (5) “What adverse impacts do you think fabricated health videos will have?” This study adopted semi-structured face-to-face interviews. The interviews were arranged in a quiet office and conducted individually to improve the quality of the data. Prior to the formal interview, the researcher described the topic and purpose of the study to the interviewees and informed them that the interviews would need to be audio recorded. The interviewees signed an informed consent form that allowed the interviews to be audio recorded. During the interviews, interviewers observed and recorded nonverbal behaviors, such as the expressions and actions of the interviewees, and confirmed their views. The average interview duration was approximately 24 min. When problems were encountered during the interviews, such as the interviewees' difficulty in recalling whether they had the experience of watching the short videos' health disinformation, or whether the interviewees’ answers to the questions were short, the researcher patiently provided guidance and offered the interviewees sufficient time to recall and answer the questions. After the interviews were completed, the recordings were first converted into text using Microsoft Word. The text was then perused, collated, and irrelevant content removed to improve data quality. After the content saturation test, effective documents of about 90,000 words were finally obtained, named N01 ∼ N22. The 22 documents obtained were the final samples for the qualitative research analysis.

### 2.5 Data analysis

Content analysis was used to analyze the research data. Simultaneously, NVivo 12 Plus was used to assist in the coding work of this study to standardize and normalize the analysis process. The main steps were as follows: First, the interview data with great significance were coded. Second, according to the coding similarities and differences, coding was included in the corresponding topic category. Finally, the topics were modified and reorganized to determine the final topic directory.

## 3 Results

**Fig. 1:**
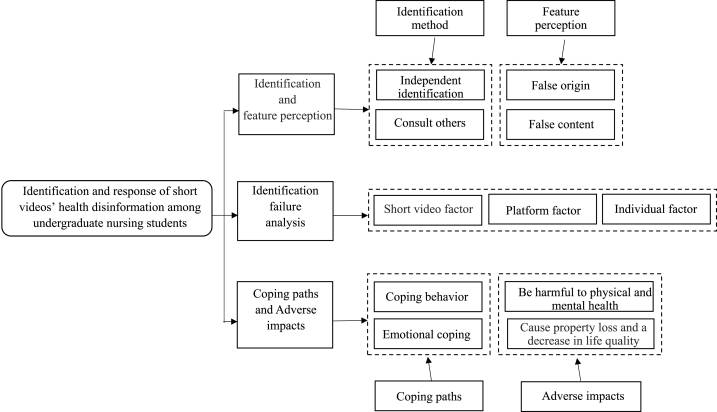
Identification and response of short videos' health disinformation among undergraduate nursing students.

This study mainly discusses the interviewees' identification and feature perception of short videos’ health disinformation, the analysis of identification failure, and the coping paths and adverse effects. The model diagram is shown in Fig. 1.

### 3.1 Identification and feature perception of short videos health disinformation

#### 3.1.1 Identification methods: Independent identification, consult others

##### 3.1.1.1 Independent identification

Although college students generally use the internet to obtain health information, they are skeptical about the short health videos. The methods for independent identification of interviewees included subjective basis identification, multi-information comparison, and their own practices. (1) Subjective identification criteria included the source of the video, reviews, and commercial promotions. The interviewees rated short videos posted by creators with authoritative sources and a large number of followers as more credible. For example, they rated short health videos posted by certified doctors, especially those with a national certification, as authentic; some interviewees would judge according to the comments; if the majority of the website's user reviews were positive, they would consider the short video to be correct; the majority of interviewees thought that short health videos with advertisements were less trustworthy [59] and they would avoid short videos with promotional links. Some interviewees believed that short videos in which most of their time was spent promoting a product were not trustworthy. (2) Some interviewees searched for relevant information on other platforms (such as Baidu, Taobao, and Weibo) for comparison, and the information with a higher frequency was regarded as true. (3) According to their circumstances, such as illness, some interviewees applied the methods introduced in the video themselves or tried the products recommended in the short video to judge their authenticity. The following interviewees described their own identification methods.

*I would definitely verify it on other platforms. I do not want to have a bad effect on myself, like allergies or something. I would definitely compare various statements on multiple platforms, see if I could find the negative effects, and finally determine whether what the video said was correct or suitable for me.* (N3)

##### 3.1.1.2 Consult others

Some interviewees would also consult their friends, classmates, or teachers when it was necessary to verify the authenticity of short videos. Interviewees perceived the content of short health videos to be accurate when the people around them perceived the content positively; otherwise, they were more likely to rate the short health video as untrustworthy. Their recognition was affected by external factors.

*Since my classmate and I are almost the same age, he may have the same purpose as me or have tried some methods in popular videos. I would discuss it with them and learn from their experience. I would trust the opinions of my classmates and friends. I sometimes consulted teachers of specialized courses.* (N2)

#### 3.1.2 Feature perception: False source, False content

##### 3.1.2.1 False origin

The false source of a short video refers to the fake name of the authority or the lack of a scientific basis. Some short health disinformation videos were spread by well-known universities to gain more people's trust. For example, “The product was developed by a university,” “Research by a university showed that …” Etc. The sources of some technical terms, data, or test reports in some short health videos were unclear, or the experiments in the videos lacked a scientific or rigorous nature.

*There were false videos that always contain unrealistic data. For example, “what percentage of this figure?” I think he compiled the data himself, without a scientific basis.* (N15)

##### 3.1.2.2 False content

Content features refer to content exaggeration and narrative ambiguity. Interviewees said that it was easy to judge the authenticity of the exaggerated content, while some interviewees believed that such short videos could mislead users. For example, some short videos’ health disinformation used quite affirmative expressions such as “no side effects,” “100 percent effective,” and “the most authentic on the Internet.” A blurred description mainly refers to a general description and a lack of pertinence and accuracy in some short videos. Interviewees rated videos with one-sided narrative content and ambiguous semantics as low-quality.

*What is not credible should be those who say that they can lose a lot of weight in three days or a few days, and that many years of rheumatism after using this medicine can be cured in a week. It is similar to those healthcare products, because I have seen one before saying that compressed sugar tablets can control blood pressure. I think this exaggerated statement is false.* (N20)

### 3.2 Identification failure analysis of short videos’ health disinformation

#### 3.2.1 Short video factors: Short video incitement, Short video confusion, Short video catering, Short video dissemination

##### 3.2.1.1 Short video incitement

Incitement was one of the reasons interviewees were cheated. When interviewees viewed short videos on health disinformation, the high provocation of the short videos made them emotionally resonate with the video creator. For example, some short videos' health disinformation would attract users with titles such as “I'm shocked,” “emergency news,” and “a student must see,” or publishers would use exaggerated words to enhance the video effect and cheat users. At the same time, the tone, rhythm, exaggerated expression, and background music of the publisher enhanced the appeal and transmission effect of the short videos, making the interviewees closely follow the video rhythm, which, to some extent, limited their ability to carefully identify the authenticity of the short video.

*Maybe it is because people have no discrimination ability for this kind of video and the content was expressed truthfully. His tone was also very exciting, so people could trust him. His tone of voice was touching, and some of the things he said online were exaggerated. This would make you not trust it, but trust it again.* (N12)

##### 3.2.1.2 Short video confusion

Confusion is another reason why people are unable to identify health disinformation through short videos. Some short videos' health disinformation had images processed by software to enhance the propaganda effect. Random editing, patchwork, and video filters also enhanced the confusion in short videos. Additionally, some short videos’ health disinformation included the words “personal testing is useful” at the beginning of the video, intending to induce users to spread it without considering its authenticity. Some video publishers could add their own before-and-after comparison pictures, detection reports, etc. However, interviewees said that the pictures might also be processed, and the detection report might be forged, which would further deepen video confusion, making it difficult for users to identify the authenticity of the video.

*At the beginning of the video, they could talk about their own feelings, so I thought it was very good. He said it was evaluated and then promoted to everyone, but he need not have generalized, and I thought it was brainwashing others.* (N11)

##### 3.2.1.3 Short video catering

The originality, interactivity, and sociability of short videos provide the younger generation with a user-friendly experience and a sense of engagement when seeking health information [60]. Information presented in short videos is easier to understand and remember than in plain text [61]. In addition, short video creators will pay more attention to the needs of users and grasp the psychological state of users to gain their recognition and attention more easily. When interviewees encounter short videos’ health disinformation that caters to their needs, such as weight loss, and have little knowledge of the content, they may not be able to effectively identify disinformation.

*I wanted to lose weight for a while, and I searched for some short videos of weight loss, among which a blogger recommended a weight loss product. She said that this product would be effective after a week’s use, and the price of this product was acceptable to me, which met my needs very well. I placed an order to buy it, only to find that it was not as effective as the video said.* (N22)

##### 3.2.1.4 Short video dissemination

In this age of social media, most sites are free to access, and any user can view and spread information. Functions such as “like” and “share” on short video platforms will also boost the spread of short videos. On social media platforms, people are more likely to repost information that they trust to be true, but that itself may not be objectively true [62], and health information spreads faster and more widely than accurate information [36,63]. Currently, short video publishers can increase video traffic by promoting the platform and further expanding the spread of short videos. Once inaccurate information begins to circulate, it becomes difficult to contain or mitigate its effects. Disinformation spreads because it is driven by emotions, especially fear, and it is difficult to combat emotions using facts [64]. The widespread use of health disinformation in short videos can mislead more users.

*The main reason I was misled was because those videos had high click-throughs and a high number of likes, and their comments below were very good. I thought short videos trusted by many people might be authentic, but I finally found them to be false. Some video creators would find people to praise and comment on videos on purpose.* (N21)

#### 3.2.2 Platform factors: Short video platform audit mechanism, Short video algorithm recommendation mechanism

##### 3.2.2.1 Short video platform audit mechanism

As everyone can publish information on social media platforms, most information lacks formal censorship. Interviewees said that some of the short videos’ health disinformation involved multiple subjects, which made platform reviews more difficult. The lack of platform audit capabilities is a major challenge for platforms.

*In fact, sometimes I think that there are so many short videos released every day, and many short videos involve many aspects. Though some short videos' health disinformation made people feel right, they were actually exposed as false in the end.* (N8)

##### 3.2.2.2 Short video algorithm recommendation mechanism

The algorithm recommendation mechanism of the short video platform would offer more health videos' disinformation of similar types to the interviewees, and users may ignore their judgment of the content, leading to interviewees being influenced by the videos' health disinformation. Such personalized recommendations have also become the basis for users’ emotional resonance on the Internet.

*For example, if a short video was fake but I did not recognize it, so, I thought it was real. The platform might recommend the same short video to me all the time, so that I would never know that I was cheated.* (N9)

#### 3.2.3 Self-factors: Low health literacy, eager mentality, herd mentality, trust mechanism

##### 3.2.3.1 Low health literacy

People with higher health literacy can understand internet health information more easily; therefore, they are not likely to be confused by disinformation. However, people with low health literacy will easily trust internet health information, and it is difficult for them to judge the reliability of the information [65]. Some interviewees said that if they did not know enough about the content of a short video, their opinions would be more favorable toward the video.

*First of all, people probably did not hear about it, so they did not know it was fake. As they lacked understanding or their cultural knowledge level was low, they would easily trust what they saw in the video.* (N2)

##### 3.2.3.2 Eager mentality

When interviewees were eager to achieve their goals, they paid more attention to the methods in short videos, recommended health products, and end results. Meanwhile, interviewees who paid too much attention to the effect relaxed their vigilance and ignored judging the authenticity and reliability of short videos’ health disinformation. Eager interviewees might fall into the “trap” of health disinformation eventually.

*Sometimes I was blind; for example, I had acne on my face and I was anxious to get rid of it first. I used many useless methods before, and I was very anxious. I might have trusted the statement in the video immediately, and I thought it was authentic subjectively.* (N13)

#### 3.2.4 Herd mentality

Owners of short videos with health disinformation can spread their videos using underhanded methods by presenting the effects of certain products to users. Interviewees displayed a herd mentality when a short video met their needs or when they saw good renderings or positive feedback from users in the comments. Users with a herd mentality are more likely to accept suggestions from others, and their ability to rationally discriminate information is weakened, eventually leading to recognition failure.

*There are also those who blindly follow the trend, such as some popular products. They might feel that people around them bought too much, but that product did not suit them or the propaganda might also be fake. We should be more careful while considering this aspect because things that are suitable for us are the most important.* (N22)

##### 3.2.4.1 Trust mechanism

Some interviewees paid attention to bloggers on short video platforms and trusted the health videos or products recommended by them. The finding that interviewees were more concerned about the identity of the authors than the content and quality of the video is consistent with previous research [66].

*Just like blindly following stars, when I saw the recommended products by a blogger, I would blindly buy from the blogger because he was my favorite blogger and I trusted the products. It may have been because I liked the blogger himself and then liked his words more. I have been following him for a long time. Can he lie to me?* (N16)

### 3.3 The coping strategies and adverse impacts of short videos’ health disinformation

#### 3.3.1 Individual coping mechanisms: coping behavior, emotional coping

##### 3.3.1.1 Coping behavior

When the interviewees watched short videos on health disinformation, they displayed several coping behaviors. On the Internet, they commented and reported on the short videos and canceled the publisher's attention. However, they also stated that reports and comments would not stop the spread of short videos' health disinformation. Some interviewees clicked “not interested” to reduce the number of similar videos. In reality, they warned their friends and relatives to prevent them from being cheated.

*I mostly told people around me, and then I did not really comment on it. Because your comments would be deleted. The main thing is to tell people around you not to trust such videos, so as not to cause bad effects.* (N15)

##### 3.3.1.2 Emotional coping

Users who obtain health information from social media can reduce negative emotions [67]; however, when they are exposed to short videos, health disinformation affects their emotions and trust in relevant health information. In this study, interviewees' emotions were divided into emotional responses that they identified and those that did not identify short videos’ health disinformation.

First, when the interviewees identified the short videos' health disinformation, most were upset. They thought that short videos' health disinformation would waste their time and energy. Second, when short videos' health disinformation was widespread and gained public recognition, interviewees were shocked by the prevalence of short videos' health disinformation and the attitudes of other users. Finally, interviewees stated that the widespread spread of health disinformation from short videos would mislead more users and even cause serious consequences. Interviewees could not stop the spread of short videos’ health disinformation through their actions, and most interviewees felt helpless.

*Sometimes I feel that the video looks very fake on the surface. They may cheat more people to get traffic, and they feel very immoral. However, I was so helpless that I had no way to make others trust me, and my comments on the video might be deleted by the video’s author.* (N11)

The second category was the emotional coping of interviewees who did not recognize the short videos’ health disinformation. Some interviewees were confused when they saw different answers and lacked discernment. For interviewees who did not identify with or act against health disinformation, most of their emotions ranged from expectation to disappointment, showing disgust and anger at health disinformation.

*After I was cheated, I was initially very angry because of the influence of such false advertising, and I was very frustrated. I may not trust such things in the future.* (N16)

#### 3.3.2 Adverse impacts of short videos’ health disinformation: Harmful to physical and mental health, it can cause property loss and lead to a decrease in life quality

##### 3.3.2.1 Be harmful to physical and mental health

Extensive, hard-to-tell information makes it difficult for people to search for reliable and trustworthy information. First, health disinformation in short videos can lead people to form wrong health opinions, which can lead them to adopt wrong health behaviors or miss the optimum treatment period to treat diseases, ultimately harming the body. Second, the use of false health products may cause unnecessary harm. Finally, users who do not reach their final and expected goal may become more anxious and may even suffer from mental illnesses.

*If people use the dubious health care products promoted in the videos, it may lead to many side effects. If you do not use health care products, you will not suffer from this. Some people may also use the wrong weight-loss methods and end up with muscle strain or malnutrition, which may make them more pessimistic. Some people may not pay attention to the disease because of the wrong information in the video and finally delay proper and credible treatment.* (N7)

##### 3.3.2.2 Can cause property loss and lead to a decrease in life quality

On short video platforms, some unscrupulous merchants or vloggers seek profits through excessive packaging or hyped health products. This marketing model encourages users to consume through entertainment and information dissemination, thus causing financial losses for users. When users fall ill and need to be hospitalized because they trusted the health disinformation in the short videos, they lose money, and their normal lives may be disrupted. Besides, some of the absolute, pseudoscientific descriptions in the short videos’ health disinformation may make the interviewees live in a state of panic, which ultimately may reduce the quality of life.

*Many fake health products are sold online, and they may be very expensive. Actually, they have no effect. Sometimes they may cause problems in life, or cause problems in learning. I may feel dissatisfied and irritated and become particularly disappointed with this video. Then, I will be very unhappy when I do other things.* (N22)

## 4 Discussion

### 4.1 Major findings

This study mainly explored interviewees' identification methods, failure factors, response paths, and the negative effects of short videos' health disinformation. By exploring the identification methods of undergraduate nursing students and identifying the reasons for failure, we can further understand their views on short videos’ health disinformation. Thus, we can provide countermeasures for the prevention of health disinformation in short videos. These negative effects also indicate the importance of preventing disinformation.

The interviewees felt that experts had a higher level of competence and proficiency; therefore, professional sources could effectively improve the credibility of the videos. Videos from healthcare professionals have higher reliability and quality than those posted by individuals [68]. Second, the practices of some interviewees may have increased the risk of negative effects. In addition, short video reviews are an important identification basis for interviewees; however, fake reviews can mislead them from making correct decisions [69]. Finally, the majority of interviewees searched for similar information to compare and trust that the information that appeared more frequently and was highly recognized by the audience was true. The more sources of information, the more opportunities individuals have to acquire knowledge and, ultimately, the more likely they are to effectively discern disinformation from facts [70]. However, some studies demonstrate that videos with more likes, shares, and comments are of lower quality, and TikTok viewers are unable to distinguish between high- and low-quality videos [71]. Interviewees’ partial screening methods or identification basis for short health videos also have drawbacks. Science-based screening methods are the key to improving the accuracy of video screening.

Various factors could have made the interviewees fail to identify short health videos, including the short video itself, the platforms, and individuals. First, some short videos' health disinformation fits the psychological expectations and stance of the interviewees [63], and misleading videos are more influential. Disinformation reduces interviewees' ability to recognize the accuracy of the information content. The gap between high- and low-quality health information in short videos is due to objective and subjective factors in healthcare, which make it difficult for the public to differentiate between accurate and inaccurate information in today's intricate social media environments [72]. Second, videos posted on social media are often ranked based on popularity, and top-ranked videos may not be peer-reviewed [73]. Additionally, interviewees indicated that they would trust goods and opinions recommended by their favorite video creators or people they knew well and were less likely to judge their credibility. This supports the findings of previous studies [74,75]. However, some interviewees reported that they also experienced health disinformation, and this blind trust may also be a factor in interviewees' failure to identify health disinformation in short videos. Finally, if short videos' health disinformation were widely disseminated, the repetition of the disinformation would result in significantly higher levels of trust, possibly because the repetition deepens the impression of the disinformation [75,76]. A combination of low personal health literacy and psycho-emotional and external factors caused interviewees to fail to recognize the health disinformation provided by short videos.

This study also analyzed interviewees' coping paths and the negative effects of short videos on health disinformation. This study explored what interviewees do and how their emotions change when confronted with short videos containing health disinformation, where interviewees report such videos and keep people around them informed. Health disinformation is narrated in a personal, negative, and opinionated tone that evokes fear, anxiety, and distrust [[77], [78], [79]]. Fearful and skeptical people may be more vulnerable to disinformation [24]. The emotional responses of the interviewees in this study were classified into two categories: identifying and not identifying health disinformation in short videos. The former exhibited annoyance, surprise, and helplessness. The latter displayed confusion, anger, and anxiety after being affected by the short videos’ health disinformation.

By exploring the interviewees' identification and coping behaviors regarding short videos' health disinformation, it was concluded that TikTok's governance of short videos' health disinformation may be inadequate. First, TikTok may lack regulations, leading users to be exposed to health disinformation from short videos. Second, although TikTok's algorithmic recommendation can accurately recommend the content in which users are interested, it also makes it easy for users to browse more similar disinformation and trust the disinformation. Finally, although TikTok established a public complaint and reporting platform and said that it would accept and handle complaints in a timely manner, some interviewees said that they did not receive any response from the platform when reporting short videos of health disinformation.

Preventing health disinformation from short videos requires the joint efforts of creators, relevant departments, platforms, and individuals. (1) Short video creators should enhance their personal moral cultivation, ensure the scientific nature and accuracy of short health videos, and spread short video health information only for personal interests. (2) First, relevant departments and platforms should formulate more laws, regulations, and anti-violation measures to curb the release of such health videos. Second, the platform should cooperate with professional departments to strengthen the screening of short videos, provide feedback, and guide public opinion in a timely, comprehensive, and effective manner. Furthermore, the platform should further optimize the algorithm recommendation mechanism to improve the quality of short videos and offer users a more comprehensive and accurate understanding of the content. Finally, effective health-promotion videos should strike a balance between scientific accuracy, popularity, duration, and ease of understanding [80]. It is necessary to consider the psychological state of users and provide alternative explanations for incorrect information in the process of correction [81]; otherwise, it will be difficult to achieve the expected effect of persuasion. Healthcare professionals, such as doctors and therapists, should verify the suitability and quality of the video and make recommendations to ensure that the patient receives appropriate information [82,83]. (3) Users should improve their health literacy, remain alert, and rationally discriminate between short videos. Health information should be obtained from authoritative and scientific platforms to prevent short videos' health disinformation. Simultaneously, users should rationally analyze the influence of external factors and be wary of video comments and suggestions given by others. Only through the joint participation of multiple parties can the proliferation of short videos’ health disinformation be effectively curtailed and the advantages of short health videos be better brought into play.

Previous research, which surveyed 150 Americans across the region with the aim of assessing individuals' willingness to share non-evidence-based YouTube videos about boosting the immune system, has shown that information literacy and science literacy are associated with low susceptibility to disinformation and less dissemination of disinformation [84]. Previous reviews have systematically evaluated published empirical studies that investigate individual differences in susceptibility to health disinformation, suggesting that conspiracy theories, religious beliefs, conservative ideology, and Conservative Party identification are associated with greater susceptibility to health disinformation [85]. One study conducted focus group interviews with 21 cancer patients to explore how they obtain disinformation about cancer and their perceptions of disinformation, and the results suggest that patients receive irrational and unreliable information from the media and need appropriate interventions from specialists and the government [86]. Using a qualitative research methodology, this study aimed to explore the identification strategies, coping paths, and impact of short videos' health disinformation on undergraduate nursing students. Unlike previous studies, undergraduate nursing students' exposure to disinformation was not only related to health literacy but also to their eagerness to watch short videos on health disinformation, herd mentality, the platform's vetting and algorithmic recommendation mechanism, as well as the incendiary, confusing, catering, and disseminating nature of the short videos. Additionally, this study investigated how undergraduate nursing students responded to and were affected by short videos' health disinformation.

### 4.2 Limitations

The limitations of this study are summarized as follows: (1) Our interviewees were from the same province and were similar in age. The qualitative data provided do not necessarily reflect representative findings. To address this limitation, future research can choose different majors, regions, and age groups, such as the elderly, remote areas, and other vulnerable groups, to make the research results more scientific and representative. (2) Although qualitative research can provide a deeper understanding of nursing students' identification with and responses to health disinformation, this study has some limitations. We faced greater challenges in analyzing the semi-structured interview data, and there was a lack of diversity in the conversation. Additionally, despite careful analysis using NVivo 12 Plus in this study, the coding remained subjective and biased owing to the involvement of researchers. To obtain higher-quality data and more scientific results, interviewers must be proficient in asking probing questions, have excellent language organization skills, and politely and tactfully guide the interviewee to answer more content without causing unwanted topic deviations. Researchers should also have excellent data analysis skills to avoid the influence of subjective factors on research results as much as possible. (3) This study adopted a qualitative research methodology. Future studies should adopt more sensitive and diverse research methods to explore health disinformation from short videos. (4) This study mainly explored the influence of short videos’ health disinformation on individuals. Future studies should explore their influence on individuals and society.

## 5 Conclusions

This study mainly explored undergraduate nursing students' identification and feature perception of short videos' health disinformation, failure factors in recognizing short videos' health disinformation, coping paths, and the adverse impacts of short videos' health disinformation. Interviewees will apply screening methods and bases that they consider reliable, including independent identification, consultation with others, and the sources and content of short health videos. The interviewees judged authenticity based on video comments, comparisons, or the source of the video. The interviewees thought that the information posted by their favorite video creators was correct and accurate or that short videos posted by well-known short video creators were more reliable. Additionally, the interviewees deemed that the information posted by doctors and experts certified as authoritative was more reliable; in contrast, short videos pretending to be authoritative and with exaggerated content are more likely to be false. However, certain screening methods are not always reliable. Interviewees may fail to recognize short videos that include health disinformation because of the platforms, short videos, and personal factors. The incendiary, confusing, pandering, and spreading nature of short videos is related to interviewees' vulnerability to disinformation. The platform's lack of vetting and algorithmic recommendation mechanisms exposed interviewees to short videos' health disinformation. Individuals' health literacy, eagerness, herd mentality, and blind trust may cause interviewees to neglect the objective, rational, and comprehensive identification of short video content. In the face of short videos' health disinformation, interviewees implemented behaviors, emotional responses, and emotional change processes. Given that health disinformation in short videos leads to health damage, property loss, and other negative effects, its governance and prevention require multiparty efforts.

## Ethics approval and consent to participate

This study conforms to the principles outlined in the Declaration of Helsinki and has been approved by the Ethics Committee from Xinyang Normal University (Study ID: XFEC-2022-21). Written consent was obtained from all participant in this study.

## Consent for publication

Not applicable in the declarations section.

## Availability of data and materials

Our data were collected for the research group and are not publicly available.

## Funding

This work was supported by the Humanities and Social Sciences Project of 10.13039/501100009101Henan Provincial Department of Education (No. 2023-ZZJH-057), the 10.13039/501100003819Hubei Provincial Natural Science Foundation (No. 2022CFD073), the Soft Science Project of Xinyang (No. 20230043), the Teacher Education Curriculum Reform Research Project in Henan Province (No. 2023-JSJYZD-018), the 10.13039/501100011788Philosophy and Social Science Planning Project of Xinyang City (No. 2023SH008), the 10.13039/501100002703Jiangsu University Philosophy and Social Science Research General Project (2022SJYB0586), the 10.13039/100006831Air Force Medical University New Flight Program and the 10.13039/501100012338Xinyang Normal University University Student Scientific Research Fund Project (No. 2022-DXS-157 and No. 2022-DXS-160).

## Data availability

Has data associated with your study been deposited into a publicly available repository?

None. Data included in article/suppmaterial/referenced in article.

## CRediT authorship contribution statement

**Ming Yang:** Writing – original draft, Methodology, Investigation. **Wanyu Huang:** Writing – original draft, Project administration, Methodology, Investigation. **Meiyu Shen:** Writing – review & editing, Project administration, Methodology, Investigation, Funding acquisition, Data curation. **Juan Du:** Writing – review & editing, Project administration, Methodology, Investigation. **Linlin Wang:** Writing – review & editing, Project administration, Funding acquisition, Formal analysis, Data curation, Conceptualization. **Yin Zhang:** Project administration, Data curation. **Qingshan Xia:** Writing – review & editing, Project administration. **Jingying Yang:** Writing – review & editing, Writing – original draft, Project administration, Methodology, Investigation. **Yingjie Fu:** Writing – review & editing, Project administration, Methodology, Formal analysis, Data curation. **Qiyue Mao:** Writing – review & editing, Supervision, Project administration, Methodology. **Minghao Pan:** Writing – review & editing, Writing – original draft, Visualization, Validation, Supervision, Resources, Project administration, Methodology, Investigation, Funding acquisition, Data curation. **Zheng Huangfu:** Writing – review & editing, Writing – original draft. **Fan Wang:** Writing – review & editing. **Wei Zhu:** Writing – review & editing, Project administration.

## Declaration of competing interest

The authors declare that they have no known competing financial interests or personal relationships that could have appeared to influence the work reported in this paper.

## References

[1] McBriar J.D. (2022). #Neurosurgery: a cross-sectional analysis of neurosurgical content on TikTok. World Neurosurg X.

[2] (2023/5/28). The 51th China statistical report on internet development[EB/OL]. http://www.cnnic.com.cn/IDR/.

[3] Yilmaz F.S., Kudsioglu T. (2020). Evaluation of the reliability, utility, and quality of the information in cardiopulmonary resuscitation videos shared on Open access video sharing platform YouTube. Australas Emerg Care.

[4] Song S. (2022). Serious information in hedonic social applications: affordances, self-determination and health information adoption in TikTok. J. Doc..

[5] Kamel Boulos M.N., Giustini D.M., Wheeler S. (2016). Future Internet.

[6] Chen K. (2022). Assessing the quality of hearing aids-related videos on TikTok. Front. Public Health.

[7] Househ M., Borycki E., Kushniruk A. (2014). Empowering patients through social media: the benefits and challenges. Health Inf. J..

[8] Han J., Shi Y., Ma H. (2022). Assessment of videos related to lung nodules in China. Front Surg.

[9] Kong W. (2021). TikTok as a health information source: assessment of the quality of information in diabetes-related videos. J. Med. Internet Res..

[10] Moon H., Lee G.H. (2020). Evaluation of Korean-language COVID-19-related medical information on YouTube: cross-sectional infodemiology study. J. Med. Internet Res..

[11] Li H.O. (2020). YouTube as a source of information on COVID-19: a pandemic of misinformation?. BMJ Glob. Health.

[12] Chidambaram S. (2022). Misinformation about the human gut microbiome in YouTube videos: cross-sectional study. JMIR Form Res.

[13] Mueller S.M. (2020). Fiction, falsehoods, and few facts: cross-sectional study on the content-related quality of atopic eczema-related videos on YouTube. J. Med. Internet Res..

[14] Fillon M. (2022). The social media cancer misinformation conundrum. CA Cancer J Clin.

[15] Adebesin F. (2023). The role of social media in health misinformation and disinformation during the COVID-19 pandemic: bibliometric analysis. JMIR Infodemiology.

[16] Lazer D. (2018). The science of fake news. Science.

[17] (2024). Information Disorder: toward an Interdisciplinary Framework for Research and Policy Making.

[18] Allington D. (2021). Health-protective behaviour, social media usage and conspiracy belief during the COVID-19 public health emergency. Psychol. Med..

[19] Stewart R. (2022). The importance of social media users' responses in tackling digital COVID-19 misinformation in Africa. Digit Health.

[20] Pandey A. (2010). YouTube as a source of information on the H1N1 influenza pandemic. Am. J. Prev. Med..

[21] Langford B. (2021). YouTube as a source of medical information about spinal cord stimulation. Neuromodulation.

[22] Zhang X. (2022). Quality of online video resources concerning patient education for neck pain: a YouTube-based quality-control study. Front. Public Health.

[23] Grimes D.R. (2020). Health disinformation & social media: the crucial role of information hygiene in mitigating conspiracy theory and infodemics. EMBO Rep..

[24] Wang Y. (2019). Systematic literature review on the spread of health-related misinformation on social media. Soc. Sci. Med..

[25] Broniatowski D.A. (2018). Weaponized health communication: twitter bots and Russian trolls amplify the vaccine debate. Am J Public Health.

[26] Wang Y. (2019). Systematic literature review on the spread of health-related misinformation on social media. Soc. Sci. Med..

[27] Squiers L. (2012). The health literacy skills framework. J. Health Commun..

[28] Rajah R. (2018). The perspective of healthcare providers and patients on health literacy: a systematic review of the quantitative and qualitative studies. Perspect Public Health.

[29] Moretti V. (2023). A web tool to help counter the spread of misinformation and fake news: pre-post study among medical students to increase digital health literacy. JMIR Med Educ.

[30] Madathil K.C. (2015). Healthcare information on YouTube: a systematic review. Health Inf. J..

[31] Rodriguez H.A. (2018). Viewer discretion advised: is YouTube a friend or foe in surgical education?. Surg. Endosc..

[32] Boudewyns V. (2021). Experimental evidence of consumer and physician detection and rejection of misleading prescription drug website content. Res. Soc. Adm. Pharm..

[33] Diez R.A. (2012). Conceptual approaches to the study of health disparities. Annu. Rev. Publ. Health.

[34] Southwell B.G. (2023). Health misinformation exposure and health disparities: observations and opportunities. Annu. Rev. Publ. Health.

[35] Desai A.N. (2022). Misinformation and disinformation: the potential disadvantages of social media in infectious disease and how to combat them. Clin. Infect. Dis..

[36] Sylvia C.W., Gaysynsky A., Cappella J.N. (2020). Where we go from here: health misinformation on social media. Am J Public Health.

[37] Rolls K., Massey D. (2021). Social media is a source of health-related misinformation. Evid. Base Nurs..

[38] Toth G., Savastano L., Jagadeesan B.D. (2021). The social media conundrum. J. Neurointerventional Surg..

[39] Fulone I. (2022). Improving the adherence to COVID-19 preventive measures in the community: evidence brief for policy. Front. Public Health.

[40] Song S., Zhang Y., Yu B. (2021). Interventions to support consumer evaluation of online health information credibility: a scoping review. Int J Med Inform.

[41] Tan S.S., Goonawardene N. (2017). Internet health information seeking and the patient-physician relationship: a systematic review. J. Med. Internet Res..

[42] Lin X., Kishore R. (2021). Social media-enabled healthcare: a conceptual model of social media affordances, online social support, and health behaviors and outcomes. Technol. Forecast. Soc. Change.

[43] de Albuquerque V.M.M. (2021). The relationship between the COVID-19 pandemic and vaccine hesitancy: a scoping review of literature until August 2021. Front. Public Health.

[44] Razai M.S. (2021). Covid-19 vaccine hesitancy among ethnic minority groups. BMJ.

[45] Gisondi M.A. (2022). A deadly infodemic: social media and the power of COVID-19 misinformation. J. Med. Internet Res..

[46] Soltaninejad K. (2020). Methanol mass poisoning outbreak: a consequence of COVID-19 pandemic and misleading messages on social media. Int. J. Occup. Environ. Med..

[47] Fraticelli L. (2021). Characterizing the content related to oral health education on TikTok. Int. J. Environ. Res. Publ. Health.

[48] Kaur H. (2021). A proposed sentiment analysis deep learning algorithm for analyzing COVID-19 tweets. Inf. Syst. Front.

[49] Madni H.A. (2023). Electronics.

[50] Moon H., Lee G.H. (2020). Evaluation of Korean-language COVID-19-related medical information on YouTube: cross-sectional infodemiology study. J. Med. Internet Res..

[51] Rubin R. (2022). When physicians spread unscientific information about COVID-19. JAMA.

[52] Burstin H. (2023). Identifying credible sources of health information in social media: phase 2-considerations for non-accredited nonprofit organizations, for-profit entities, and individual sources. NAM Perspect.

[53] Scherr S., Wang K. (2021). Explaining the success of social media with gratification niches: motivations behind daytime, nighttime, and active use of TikTok in China. Comput. Hum. Behav..

[54] Experts grade Facebook, TikTok, Twitter (2022).

[55] Wang R., Yang F., Haigh M.M. (2017). Let me take a selfie: exploring the psychological effects of posting and viewing selfies and groupies on social media. Telematics Inf..

[56] Dhir A., Tsai C. (2017). Understanding the relationship between intensity and gratifications of Facebook use among adolescents and young adults. Telematics Inf..

[57] Pop L.M. (2021). Gender differences in healthy lifestyle, body consciousness, and the use of social networks among medical students. Medicina (Kaunas).

[58] Sharma S., Oli N., Thapa B. (2019). Electronic health-literacy skills among nursing students. Adv. Med. Educ. Pract..

[59] Kerr C. (2006). Internet interventions for long-term conditions: patient and caregiver quality criteria. J. Med. Internet Res..

[60] Song S. (2021). Short video apps as a health information source: an investigation of affordances, user experience and users' intention to continue the use of TikTok. Internet Res..

[61] Rus H.M., Cameron L.D. (2016). Health communication in social media: message features predicting user engagement on diabetes-related Facebook pages. Ann. Behav. Med..

[62] Pennycook G., Rand D.G. (2019). Lazy, not biased: susceptibility to partisan fake news is better explained by lack of reasoning than by motivated reasoning. Cognition.

[63] Vosoughi S., Roy D., Aral S. (2018). The spread of true and false news online. Science.

[64] Zucker H.A. (2020). Tackling online misinformation: a critical component of effective public health response in the 21st century. Am J Public Health.

[65] Diviani N. (2015). Low health literacy and evaluation of online health information: a systematic review of the literature. J. Med. Internet Res..

[66] Liang J. (2022). Quality and audience engagement of takotsubo syndrome-related videos on TikTok: content analysis. J. Med. Internet Res..

[67] Palsdottir A. (2014). Preferences in the use of social media for seeking and communicating health and lifestyle information. Information Research An International Electronic Journal.

[68] Pan P. (2020). Xigua video as a source of information on breast cancer: content analysis. J. Med. Internet Res..

[69] Mukherjee A. (2013).

[70] Zhou J. (2020). Health perceptions and misconceptions regarding COVID-19 in China: online survey study. J. Med. Internet Res..

[71] Sun F., Zheng S., Wu J. (2023). Quality of information in gallstone disease videos on TikTok: cross-sectional study. J. Med. Internet Res..

[72] Osman W. (2022). Is YouTube a reliable source of health-related information? A systematic review. BMC Med. Educ..

[73] Hu R.H. (2022). Quality and accuracy of gastric cancer related videos in social media videos platforms. BMC Publ. Health.

[74] Sbaffi L., Rowley J. (2017). Trust and credibility in web-based health information: a review and agenda for future research. J. Med. Internet Res..

[75] Lewandowsky S. (2012). Misinformation and its correction: continued influence and successful debiasing. Psychol. Sci. Publ. Interest.

[76] Swire B., Ecker U.K.H., Lewandowsky S. (2017). The role of familiarity in correcting inaccurate information. J. Exp. Psychol. Learn. Mem. Cognit..

[77] Panatto D. (2018). A comprehensive analysis of Italian web pages mentioning squalene-based influenza vaccine adjuvants reveals a high prevalence of misinformation. Hum. Vaccines Immunother..

[78] Porat T. (2019). Content and source analysis of popular tweets following a recent case of diphtheria in Spain. Eur. J. Publ. Health.

[79] Bessi A. (2015). Trend of narratives in the age of misinformation. PLoS One.

[80] Gong X. (2023). TikTok video as a health education source of information on heart failure in China: a content analysis. Front. Public Health.

[81] Ecker U.K., Lewandowsky S., Tang D.T. (2010). Explicit warnings reduce but do not eliminate the continued influence of misinformation. Mem. Cognit..

[82] Chang M.C., Park D. (2021). YouTube as a source of patient information regarding exercises and compensated maneuvers for dysphagia. Healthcare (Basel).

[83] Atci A. (2019). Quality and reliability of the information on YouTube Videos about Botox injection on spasticity. Rom. J. Neurosurg..

[84] Keselman A. (2021). Factors influencing willingness to share health misinformation videos on the internet: web-based survey. J. Med. Internet Res..

[85] Nan X., Wang Y., Thier K. (2022). Why do people believe health misinformation and who is at risk? A systematic review of individual differences in susceptibility to health misinformation. Soc. Sci. Med..

[86] Kim J.H. (2022). How cancer patients get fake cancer information: from TV to YouTube, a qualitative study focusing on fenbendazole scandle. Front. Oncol..

